# Plasmonically Enhanced Schottky Photovoltaic Devices

**DOI:** 10.1038/s41598-017-14528-0

**Published:** 2017-10-27

**Authors:** M. Farhat, S. Kais, F. H. Alharbi

**Affiliations:** 1 Qatar Environment and Energy Research Institute (QEERI), Hamad Bin Khalifa University, Qatar Foundation, Doha Qatar; 2 Department of Chemistry, Department of Physics and Birck Nanotechnology Center, Purdue University, West Lafayette, IN 47907 USA; 3 College of Science and Engineering, Hamad Bin Khalifa University, Doha, Qatar

## Abstract

Solar-cells based on Schottky junctions between metals and semiconductors (without or with an intermediate insulator) are among the main possibilities towards economical photovoltaic conversion of the solar energy. This is mainly due to their structural simplicity and hence the ease of their realization. We propose here a new kind of light-harvesting devices using plasmonic nano-antenna gratings, that enhance the absorption of light over a broadband spectrum, and permit the reduction of thickness of the cell dramatically, with efficiency around 15% for 3 micrometers ultra-thin Silicon cell. We show that this technique may provide a new avenue in low cost fabrication of thin-film solar-cells.

## Introduction

Today’s solar-cells provide the largest long-term power supply for applications such as satellites and small space-vehicles^1^. As the world’s demand for energy continuously increases, many traditional sources of energy (mainly fossil fuels) will be hugely decreased over the current century. Hence, there is an urgent need to come-up with novel energy resources, relying mainly on the main earth’s natural resource, i.e. the sun. Solar-cells are, undoubtedly, the most reliable candidate to harvest the sun’s abundant energy, as they transform light directly into electricity with relatively efficient photo-conversion (unlike for example thermal energy extraction). In fact, solar-cells can provide a low-cost and a nearly pollution-free permanent supply of power^2,3^. More recently, the development of solar-cells with low-cost modular panels, concentrator systems, thin-film designs, and several innovative ideas have been extensively studied. One expects in the near-future, a substantial reduction of the costs of small to medium solar modular units and solar power plants^4,5^.

Perhaps the most straightforward way for separating charges is the metal-semiconductor junction^6^. This type of junctions, is an easily-fabricated and simple way to prepare an efficient photovoltaic barrier (or junction). It has also the advantage of being simply fabricated since only a single layer of p-doping or n-doping of the semiconductor is needed, meaning that there is no need for p-n junctions which require some practically expensive processes like the high-temperature phosphorus-diffusion step for Silicon (Si) solar-cells^7,8^. However, it should be mentioned that this type of cells does not produce the highest photo-voltages^8^. In fact, when the barrier height is greater than ~*E*_*g*_/2, with ${E}_{g}$ the energy bandgap of the semiconductor, the minority carriers are more numerous than the majority ones near the interface, resulting thus in the formation of an inversion layer. The junction becomes, under these conditions, loaded with carriers and can no longer support high photo-voltage, which reduces the efficiency of the solar-cell^6,9–13^.

In order to enhance the overall efficiency of such cells, photon management can be employed^14–20^. It can take benefit from many strategies to improve the absorption of sunlight in a cell by an assistive structure^20–24^. Two main approaches can be used, to this end. On the one hand, an assistive plasmonic (or photonic) structure can be added to help reducing reflection losses (e.g. anti-reflection gratings) due to impedance mismatch near the top-interface and to increasing the optical path of the incident light into the solar-cell^14,15,17^. On the other hand, up- or down-conversion can be employed to modify the spectral composition of sunlight to mitigate losses^19^. In the same vein, plasmonics is an emerging broad area of research, that studies exceptional optical properties of metallic two- and three-dimensional (2D and 3D, respectively) nanostructures^25^, i.e. the confinement of electromagnetic waves (SPPs, i.e. surface plasmon polaritons). During the past few years, there has been a huge interest in employing these plasmonic structures for sunlight harvesting, i.e. for example in thermophotovoltaics, thin-film solar-cells, or solar thermoelectrics, etc^15,26^. All these applications exploit the exceptional capabilities of plasmonic structures to concentrate electromagnetic energy and generate hot-electrons^27–32^. For efficiency reasons, such devices need to be ultra-broadband (optical spectrum) and insensitive to polarization and angle of incidence^33^.

In this paper, we will focus on studying the ability of a simple, yet effective plasmonic metamaterial grating structure, to be beneficial for both of the aforementioned strategies. Primarily we concentrate on solid state thin-film Schottky solar-cells. To this purpose, we will design an ultra-thin plasmonic absorber, capable of absorbing more than 90% of solar energy within a broad wavelength range (300–1300 nm). This design is promising since, it would be expected that the cost of fabricating high-quality elemental material would be much cheaper (with more industrial yield) than making a high-quality ternary or quaternary materials. Other spectral ranges can also be also considered, using novel materials, such as graphene^34,35^, for thermophotovoltaics applications^36,37^. In the first section of this work, we analyze the optical modeling and response of the plasmonic grating and show its ability to enhance the photo-generation rate and thus the short-circuit photo-current. Next, we input these results in a drift-diffusion solver (SCAPS) to obtain the electronic response of the cell, in particular, its photo-conversion efficiency, that is shown to attain 15% for some geometries. Last but not least, a descriptor model^38–40^ is used to get the effect of overall thickness of the cell on its efficiency and it is shown that this strategy can be used to realize low-cost thin solar-cells that can be used in various applications. Future optimization of the geometry and materials can enhance the efficiency even further, but this will be the scope of future publications.

## Results

### Plasmonic Schottky junction

**Figure 1:**
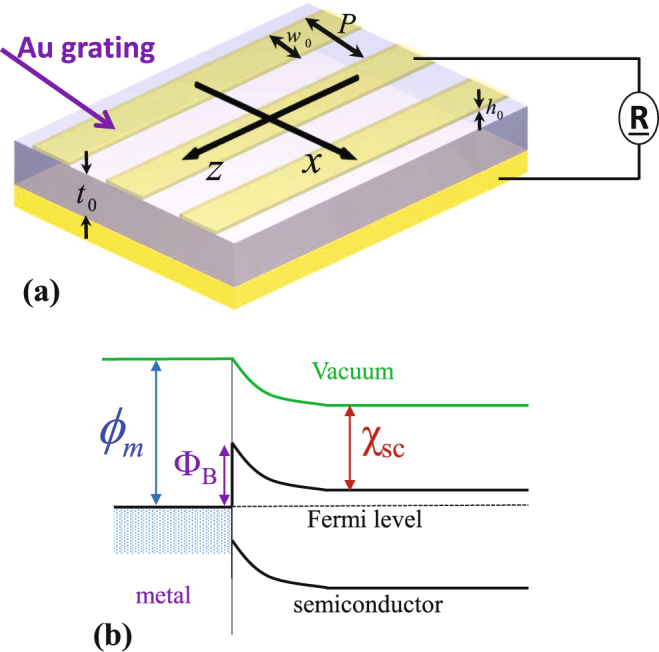
(**a**) Sketch of the proposed structure of a Schottky-based solar-cell. It is made of an active semiconductor layer sandwiched between two noble metals connected to an external load *R*. The geometrical parameters of the proposed structure are: width of the grating ${w}_{0}$, its period *P*, and its height ${h}_{0}$, and thickness of the Silicon layer ${t}_{0}$. (**b**) Band diagram for the structure described in (**a**) across the junction, showing the Schottky barrier and the different other parameters of the junction.

A metal-semiconductor contact (shown in the upper interface of Fig. 1(a)) results in a hetero-interface, i.e. widely referred to as a Schottky junction^6,10,11^, and consists of a spatial charge region with a variable free-charge support density profile formed according to the semiconductor band structure and the metal, given for the studied example in Fig. 1(b). These junctions are widely used in electronics, because of their ability to rectify currents, which results directly from a spatial charge layer on the semiconductor side of the interface. The magnitude of the Schottky barrier height is here ${{\rm{\Phi }}}_{B}$ (see Fig. 1(b)). As in this study, the semiconductor used is of n-type, one have

1 $${{\rm{\Phi }}}_{B}={\varphi }_{m}-{\chi }_{{\rm{sc}}},$$

with ${\chi }_{{\rm{sc}}}$ the affinity of the semiconductor (Silicon in this work) and ${\varphi }_{m}$ is the work function of the metal (gold, here). Similarly, the built-in potential across the depletion region is

2 $${{\rm{\Phi }}}_{{\rm{bi}}}={\varphi }_{m}-{\chi }_{{\rm{sc}}}-({E}_{c}-{E}_{F}),$$

with ${E}_{g}$, ${E}_{c}$ and ${E}_{F}$ the bandgap energy, the conduction band energy and Fermi level of Silicon, respectively. And the depletion region width can be computed and is ${\delta }_{0}=\tau \sqrt{2{\varepsilon }_{s}({\varphi }_{{\rm{bi}}}-{V}_{b})/{N}_{d}}$, with ${N}_{d}$ the doping concentration of Silicon, ${\varepsilon }_{s}$ its permittivity and $\tau$ a parameter depending on its surface properties^41^.

As well-known, within a metal-semiconductor junction, there exists two main types of current paths: minority and majority carrier density current paths (${J}_{{\rm{\min }}}$ and ${J}_{{\rm{\max }}}$, with saturation currents ${J}_{0,\min }$ and ${J}_{0,{\rm{\max }}}$, respectively). On the one hand, ${J}_{{\rm{\min }}}$ is equivalent to the current observed in classical p-n junction cells, with the major difference being that the barrier is here achieved through the depletion layer due to the metal, as opposed to doping in the aforementioned example. On the other hand, an effect specific to Schottky-junction cells (and not found for example in p-n junctions) is the presence of a majority carrier current path, the main contribution to it comes from the Schottky-Richardson emission, or thermionic emission: the carriers inside the metal can pass over the Schottky barrier if they have enough energy. This current is almost constant with regards to bias, since the barrier height (See Fig. 1(b)) is sensitive to bias (unlike the built-in potential, or the p-n junction barrier). The forward current is also present in this configuration and is due to carriers excited over the Schottky barrier. The total current is therefore $J={J}_{{\rm{\min }}}+{J}_{{\rm{maj}}}={J}_{0}(\exp (eV/{k}_{B}T)-\mathrm{1)}$, with a saturation current ${J}_{0}={J}_{\mathrm{0,}{\rm{\min }}}+{J}_{\mathrm{0,}{\rm{maj}}}$.

### Optical modeling and characterization

Figure 1(a) depicts the geometry of the structure to be considered. It is made of a gold nanostrips array on top of a ground metal (silver) contact. These two metals are separated by a relatively thin dielectric semiconductor (Silicon). The periodicity in the *x*-direction is $P=1$ *μ*m whereas the dimensions in the remaining *z*-direction are set to infinity. Here the refractive index of the dielectric Silicon layer deposited on the metal is taken from experimental data and interpolated^42^. Metals are modeled by their conductivity $\sigma$ that is extracted from experimental data (See Methods section)^43^.

**Figure 2:**
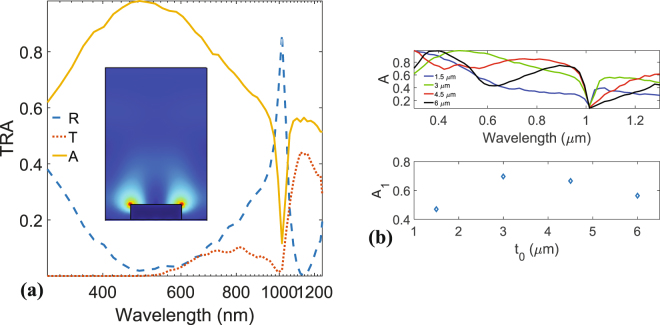
(**a**) Transmission, reflection and absorption spectra (T, R, and A) of the grating. The inset shows the magnitude of electric field at around $\lambda =1\,\mu m$. (**b**) Absorption for different thicknesses (up) and integrated absorption ($\frac{1}{{\rm{\Delta }}\lambda }{\int }_{{\lambda }_{{\rm{\min }}}}^{{\lambda }_{{\rm{\max }}}}d\lambda A(\lambda )$) versus thickness ${t}_{0}$.

The geometrical properties of the solar-cell are chosen based on experimental capabilities^21^, a filling fraction ${w}_{0}/P$ in the range 0.1 to 0.8, shown to be easier for fabrication (See Fig. 1(a)). The geometry is excited by an incident electromagnetic plane-wave, polarized as transverse magnetic (TM). COMSOL Multiphysics^44^ is used to determine the underlying physics of the absorbing device.

Figure 2(a) shows the frequency dependency of transmittance (T), reflectance (R), and absorption (A) of the plasmonic grating (of Fig. 1(a)). These results spot that great absorption enhancement is achievable for the geometry of interest. Nevertheless, by tuning the thickness of Si layer and optimizing the geometry, as well as adding the bottom metal layer, one can achieve total absorption for some frequencies; this effect is known as coherent perfect absorption and is the reciprocal equivalent of lasers^45^. In addition, it is worth mentioning that the effect of the back metal/Silicon interface on the plasmonic resonance is not significant, i.e. hybridization of the plasmon modes and creation of symmetric and antisymmetric plasmons, unless the thickness of the semiconductor is few tens of nanometers^25^. Besides, by using a thin back metal the effects on the absorption (and thus the generation) were verified to be minimal. Another possibility is the use of transparent electrodes as ohmic back contacts.

In order to investigate the robustness of this design in reducing the thickness of the solar-cell while absorbing a reasonable amount of the incident radiation, the absorption of the structure is plotted for different thicknesses ${t}_{0}$ of the Silicon layer in Fig. 2(b) (upper panel). In the lower part of Fig. 2(b) a new parameter ${A}_{1}$ is defined, that is the wavelength-integrated ($\lambda$) absorption for each thickness, i.e. $\frac{1}{{\rm{\Delta }}\lambda }{\int }_{{\lambda }_{{\rm{\min }}}}^{{\lambda }_{{\rm{\max }}}}d\lambda A(\lambda )$, with ${\rm{\Delta }}\lambda =1000$ nm, the range of wavelengths used in this work. The behavior of ${A}_{1}$ versus the thickness ${t}_{0}$, clearly shows that significant part of the electromagnetic energy (70$\%$) is absorbed by the thin plasmonic Schottky solar-cell (around 3 *μ*m in thickness of Si, and around 100 nm for the front and back metal contacts). The electric energy distribution $|{\rm{E}}|$ around the SPPs resonance is given in the inset of Fig. 2(a) and confirms the nature of the excited mode (dipolar).

**Figure 3:**
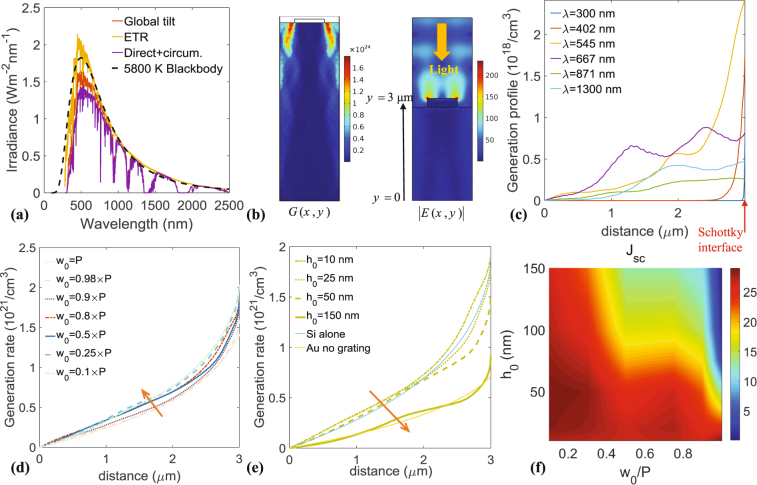
(**a**) Light spectrum generated from the sun, compared to the radiation from a blackbody at 5800 K. (**b**) (left) generation profile inside the silicon layer and (right) electric field profile. (**c**) Spectral generation ${G}_{0}(\lambda ,x=\mathrm{0,}\,y)$ for different wavelengths. (**d**,**e**) represent the light integrated generation rate (*G*) versus the depth into silicon for various periods and heights of the gold grating, respectively. (**f**) Contour plot of the short-circuit density current (${J}_{{\rm{sc}}}$) versus geometry of the grating, i.e. ${w}_{0}$ and ${h}_{0}$.

Let us move the focus now to the mechanism of electron-hole generation in the active layer of the Schottky cell. To simulate the structure and obtain realistic response, the incident electromagnetic power is given in Fig. 3(a). The solar spectral irradiance obtained experimentally (Extra terrestrial ETR, and at the surface of earth, global tilt) is plotted in the wavelength range 0–2500 nm. To simplify simulations, these experimental data are compared to the irradiance spectra from a black-body (at the temperature at the surface of the sun, i.e. 5800 K), described by

3 $${I}_{r}={(\frac{{R}_{s}}{{r}_{se}})}^{2}\frac{{\mathrm{(2}\pi )}^{2}{c}^{2}\hslash }{{\lambda }^{5}}(\frac{1}{{e}^{2\pi \hslash /(\lambda {k}_{B}T)}-1}),$$

with ${R}_{s}$ denoting the sun’s radius and ${r}_{se}$ the average sun-earth distance. The match with the measured illumination data (i.e. the NREL ASTM G173-03) is convenient^46^, and in the remaining of this work, we will use incident powers deduced from Eq. (3).

Since the main output from the optical simulations results is the generation rate (of electron-holes pairs due to incident light), one should discuss how this is computed numerically. First, the absorbed power by the plasmonic structure must be numerically computed, by solving Maxwell’s equations with finite elements technique^47^, i.e. solving through wave optics module of COMSOL, the wave equation in the frequency domain $\nabla \times (\nabla \times {\bf{E}})-{k}_{0}^{2}{\varepsilon }_{r}{\bf{E}}=0$. Then, this is done by using the divergence of the Poynting vector formula^48^, leading to

4 $${Q}_{{\rm{abs}}}=-2\pi \frac{{\rm{Im}}(\varepsilon (\lambda ))}{\lambda }|{\bf{E}}{|}^{2}.$$

It can be immediately seen from Eq. (4) that higher values of electric field result in higher absorption, confirming thus the purpose of this work to use surface plasmon polaritons (SPPs) that generally lead to great field enhancement (shown for example in the inset of Fig. 2(a)). Next, it is straightforward to link ${Q}_{{\rm{abs}}}$ to the electron-hole pairs number that are photo-generated, per unit time, as function of position (See Fig. 3(b,c)). This is done through the relation ${G}_{0}(\lambda ,x,y)=\lambda {Q}_{{\rm{abs}}}/ch$. The total generation rate is finally a spectral integration of ${G}_{0}$, i.e. $G(x,y)={\int }_{{\lambda }_{{\rm{\min }}}}^{{\lambda }_{{\rm{\max }}}}d\lambda {G}_{0}(\lambda ,x,y)$.

From Fig. 3(d) and (e), significantly enhanced charge carrier generation can be observed for the plasmonically enhanced Schottky cell, because of stronger photon absorption. The integrated generation $G(x,y)$ is plotted versus depth in the semiconductor (See Fig. 3(b) for the definition of incident light, i.e. downwards). Here, we only give G(y), and integrate over the remaining direction (See Methods section). The generation decays in exponential manner (with origin at 3 *μ*m), as expected. The role of filling fraction of the gold grating is shown in Fig. 3(d). It can be noticed that a filling of 0.5 gives good generation. The lowest generation is obtained when ${w}_{0}=P$, showing the importance of plasmonic resonance. Figure 3(e) analyses the role of height of gold grating (as shown in Fig. 1(a)). Indeed, it is shown that higher thickness results in lower generation. This can be explained by the fact that electric field decays very quickly in metals due to the plasmonic effect, and the lower the thickness the better the transmitted field into the active layer of Silicon. Values of 10 or 25 nm seem to be more suitable to enhance the generation rate. Finally a systematic calculation of the short-circuit current density ${J}_{{\rm{sc}}}$ is given versus the two parameters of the grating, i.e. ${h}_{0}$ and ${w}_{0}/P$. The result for ${J}_{{\rm{sc}}}=q\iint dx\,dy\,G(x,y)$ is shown in Fig. 3(f). The blue regions correspond to low density currents, whereas red regions correspond to higher values from the structure. It can be noticed that ranges of ${w}_{0}/P$ between 0.1 and 0.5, and ${h}_{0}$ between 10 and 50 nm, are best for high short-circuit current. The maximum of ${J}_{{\rm{sc}}}$ has coordinates (0.15, 30) that correspond to a maximum value of around 30 mA/cm^2^. We wish also to emphasize that the important parameter here is the minority carrier diffusion length. The longest path that these carriers have to travel before collection is around the thickness of Silicon layer. With our parameters, the maximum distance is around 3 *μ*m, which is far below the holes diffusion length, even in highly n-doped Silicon^49^. The normal obtained average diffusion length is around 100 *μ*m (for the level of doping we use) and the record is in the millimeter range^50,51^.

### Electronic modeling and characterization

The second step is to perform the electrical characterization using the 1D software SCAPS (Solar-Cell Capacitance Simulator)^52,53^ that solves drift-diffusion equation across the solar-cell with output from optical simulations, i.e. the profile of the generation rate $G(y)$ and by enforcing recombination through neutral defects (See section Methods for more details).

The governing equations for the electrical modeling of Schottky solar-cell can be written as the following^2,3^

5 $$\begin{array}{ccc}\frac{{\rm{\partial }}{n}_{n}(y,t)}{{\rm{\partial }}t} & = & \frac{1}{q}\frac{{\rm{\partial }}{{\bf{j}}}_{n}(y,t)}{{\rm{\partial }}y}+G(y)-U(y),\\ \frac{{\rm{\partial }}{n}_{p}(y,t)}{{\rm{\partial }}t} & = & \frac{-1}{q}\frac{{\rm{\partial }}{{\bf{j}}}_{p}(y,t)}{{\rm{\partial }}y}+G(y)-U(y),\\ {\rm{\nabla }}\cdot (\varepsilon {\rm{\nabla }}\varphi ) & = & -\rho ,\end{array}$$

with *q* denoting the unit electronic charge, $\varepsilon$ is the permittivity of Silicon, $U(y)$ and $G(y)$ are recombination and generation rate. Here *G* is taken from optical simulations (see previous Section) and for $U$ we assume neutral defects with capture cross-section of ${10}^{-14}\,{{\rm{cm}}}^{2}$ and a single energetic distribution above the valence band of 0.6 eV, i.e. the Schockley-Read-Hall mechanism^54,55^, with a uniform concentration of defects in the gap, i.e. ${N}_{t}={10}^{12}$. The charge density is $\rho =q({n}_{n}-{n}_{p}+{N}_{A}-{N}_{D})$, with ${n}_{n}$, ${n}_{p}$, ${N}_{A}$, and ${N}_{D}$ denoting the density of electrons, holes, acceptors impurities, and donor impurities, respectively, in the general case. Here we consider an n-doped Silicon layer with ${N}_{A}=0$ and ${N}_{D}={10}^{16}\,{{\rm{c}}{\rm{m}}}^{-3}$. The electron and hole current densities are

6 $$\begin{array}{c}{{\bf{j}}}_{n}=-q{\mu }_{n}{n}_{n}{\rm{\nabla }}\varphi +q{D}_{n}{\rm{\nabla }}{n}_{n},\\ {{\bf{j}}}_{p}=-q{\mu }_{p}{n}_{p}{\rm{\nabla }}\varphi -q{D}_{p}{\rm{\nabla }}{n}_{p},\end{array}$$

respectively. Here *μ* denotes the mobility and *D* the diffusion coefficient. The total current is ${\bf{j}}={{\bf{j}}}_{n}+{{\bf{j}}}_{p}$.

**Figure 4:**
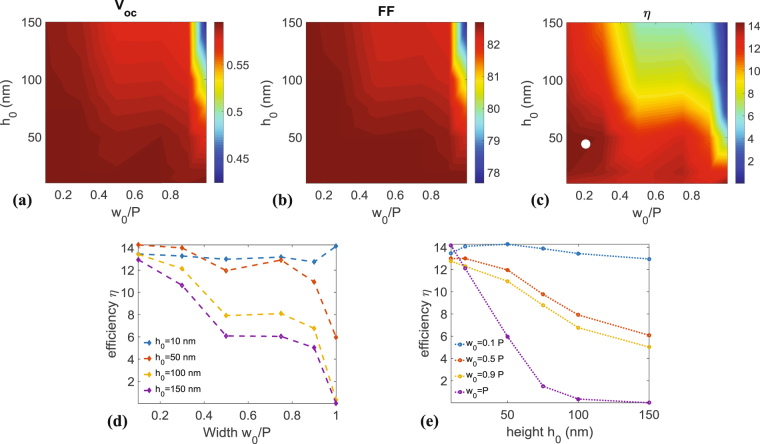
(**a**) Open-circuit voltage (${V}_{{\rm{oc}}}$) versus the width and height of the metallic grating. (**b**) Filling factor (FF) versus the width and height of the metallic grating. (**c**) Cell efficiency ($\eta$) versus the width and height of the metallic grating. Efficiency of the cell for different values of the height (**d**) and width (**e**) of the grating.

When using 1D software, the typical approach is to solve the equations in only the transversal direction (main direction of current flow through the semiconductor) and assume that there are no parameter variations in the two lateral directions (parallel to flow). Hence, the 1D results are assumed to capture the average performance of the entire solar-cell. Since the overall efficiency of all kinds of solar-cells (p-n junctions or Schottky junction cells) can be expressed as

7 $$\eta =\frac{{V}_{{\rm{oc}}}{J}_{{\rm{sc}}}FF}{{P}_{{\rm{inc}}}},$$

we analyze here the geometrical variations of *V*_oc_, *J*_sc_, and FF. *J*_sc_ was given in Fig. 3(f). Figure 4 plots ${V}_{{\rm{oc}}}$ and FF as functions of the filling fraction and height of the gold grating. The voltage values oscillate around 0.5 V (See Fig. 4(a)), attaining a maximum of 0.6 V for ${w}_{0}/P=0.1$ and ${h}_{0}=50$ nm, whereas FF attains its maximum, i.e. 82.7% for the same parameters as before (See Fig. 4(b)). The photovoltaic efficiency $\eta$, shown in Fig. 4(c), have a similar behavior, although it is more sensitive to variations in ${w}_{0}$ and ${h}_{0}$ similarly to ${J}_{{\rm{sc}}}$. The maximum value for $\eta$ highlighted by a white dot (See Fig. 4(c)) is around 14%. This is attained for ${w}_{0}/P=0.1$ and ${h}_{0}=50$ nm ($\eta =\mathrm{14.27 \% }$) and for ${w}_{0}/P=0.9$ and ${h}_{0}=10$ nm ($\eta =\mathrm{14.15 \% }$). These results show the importance of taking into account both the grating thickness and width. This can be better understood from Fig. 4(d),(e), where the efficiency is plotted versus ${w}_{0}/P$ for various values of ${h}_{0}$, and versus ${h}_{0}$ for various values of ${w}_{0}/P$, respectively. The sensitivity to variations in geometrical parameters is more evident from these figures. From Fig. 4(d), it can be seen that a thickness of 50 nm is ideal for photovoltaic conversion (around 14% that drops to 6% *w*ith increasing width *w*_0_). The case of *h*_0_ = 10 nm is slightly different, since $\eta$ does not experience any drop. This can be explained by the skin depth, i.e. ${\delta }_{0}=\lambda /\mathrm{(2}\pi \Im ({n}_{g}))$ of gold at optical frequencies, with *n*_*g*_ the refractive index of gold. In fact, the average value of *δ*_0_ is around 30 nm for the wavelength domain into consideration. Therefore, the radiation can be transmitted to the active layer where it can generate carriers, using the SPPs effect, in contrast to a thickness of 100 nm, where no radiation is transmitted for *w*_0_ = *P*, explaining the drop in $\eta$ to 0. From Fig. 4(e), it can be seen that a width of gold strips of ${w}_{0}/P=0.1$ is ideal and less sensitive to variations in the thickness *h*_0_, giving an efficiency between 13 and 14%. Increasing *w*_0_ increases the sensitivity of $\eta$ on *h*_0_, as well.

## Discussion

As stated earlier in this report, the key difference (advantage) of fabricating Schottky barrier solar-cells with respect to the p-n solar-cells fabrication is the omission of the step of phosphorous-diffusion, that generally creates the n-Silicon layer of the Silicon cell (or any other semiconductor). The elimination of this step reduces the energy cost of production of the cell by 35%^7^. The mechanism of the barrier is also different since it relies on the difference of energy bands of metal and semiconductor. The geometry of this kind of cells is also more appropriate to plasmonic enhancement, since one can just adjust the geometry and material properties of the front contact to enhance light absorption and thus generation rate and use this layer in the same time as contact. We should mention here too, that the mechanism of the cell proposed here is intrinsically different from hot-electrons based plasmonic solar-cells^7,8^ that have limited efficiency due to ultra-short lifetime of excited electrons in the metal^27,29,31^.

**Figure 5:**
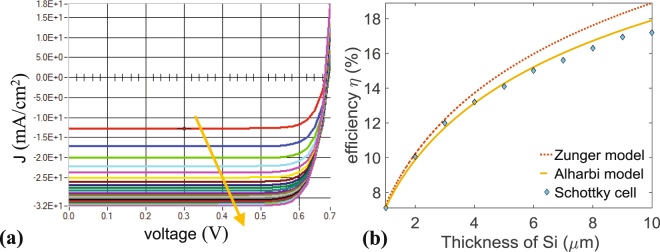
(**a**) Current-Voltage (J-V) curve for different thicknesses. The yellow arrow indicates increasing thickness of the Silicon layer (from 1 to 50 *μ*m) (**b**) Efficiency versus thickness of silicon layer deduced from the model of refs^38–40^.

In order to further analyze the enhancement due to the absorber layer’s thickness, the generation rate $G(y)$ is calculated as before for thicknesses ${t}_{0}$ ranging from 1 to 50 *μ*m, and the corresponding $J-V$ characteristic of the Schottky cell is calculated via SCAPS and is plotted in Fig. 5(a) showing the increase in ${J}_{{\rm{sc}}}$ with increasing ${t}_{0}$. The increase in ${V}_{{\rm{oc}}}$ is also present, but less remarkable. This indeed shows that ${J}_{{\rm{sc}}}$, as expected, is sensitive to ${t}_{0}$ in contrast to ${V}_{{\rm{oc}}}$. $\eta$ is proportional to ${J}_{{\rm{sc}}}$, so it increases with ${t}_{0}$, as well, as can be seen in Fig. 5(b) (diamond plot). Nonetheless, varying ${t}_{0}$ from 1 *μ*m to 10 *μ*m results in an increase of $\eta$ from 12% to only 16%, meaning that to realize a low-cost and simply constructed cell with acceptable efficiency, ultra-thin Si cell of only few micrometers is sufficient. These results are compared to the efficiencies obtained from two descriptor models with accurate estimations. The plot in dashed-line corresponds to the SLME model (spectroscopic limited maximum efficiency) first developed by Yu and zunger *et al*.^38^ while the one in continuous-line corresponds to the improved Scharber model^39^ developed by Alharbi *et al*.^40^. Both models give almost similar estimations, although the latter one provides more accurate predictions for ${J}_{{\rm{sc}}}$ and ${V}_{{\rm{oc}}}$ ^40^. The analysis of simulations shows that with the proposed plasmonically enhanced Schottky cell, the practical efficiency limit can be achieved as both models are close to the performance of the proposed Schottky cell. From these plots, one can assert that the proposed solar-cell using the plasmonic effect to enhance the generation rate can lead to ultra-thin simply constructed designs with economically respectable efficiencies. It should be added, too that the efficiency of the Schottky solar-cell (around 15%) can potentially be improved by modifying the structure so that the plasmonic resonators are electrically separated from the semiconductor. This can be achieved by placing an ultra-thin dielectric spacer between the semiconductor and the metal resonator array, i.e. a few nanometers thin-tunnel barrier, beneficial to reducing ${J}_{\mathrm{0,}{\rm{maj}}}$ (i.e. the majority density current), for the most part, without markedly decreasing ${J}_{{\rm{sc}}}$ (i.e. the short-circuit current), that implies that minority carriers cross the tunnel thanks to the insulation. Moreover, the asymmetry is enforced, since the minority carriers are driven by the electric field into the barrier, where they can be piled relatively while waiting through the tunnel. On the other hand, most carriers, are pushed out from the interface by the effect of the electric field, thus reducing their pace, which can greatly decrease their net flow^7^.

### Conclusion

We proposed an alternative methodology for designing plasmonically enhanced solar-cells. The structure made of nano-patterned plasmonic arrays permits a sizable enhancement of absorption by the Silicon layers, made as thin as 3 *μ*m. We have studied the specific example of Schottky-based solar-cells and demonstrated their efficiency. The light-trapping in these devices takes place thanks to the excitation of plasmonic modes of gold nano-antennas. These nano-antenna arrays are shown to operate better than the generally used anti-reflection layers. In fact, enhancement in the photo-generation rate was shown and was reflected in an increased photovoltaic conversion efficiency, that can attain 15% for certain geometries, which conjugated to the simplicity of fabrication, can open new avenues in low-cost photovoltaic devices with average efficiencies.

## Methods

### Finite-elements simulations

Finite-elements frequency-domain simulations were carried out using COMSOL Multiphysics software^44^ version 5.2. All simulations were for a two-dimensional array with periodicity of 1 *μ*m, with enforced periodic-boundaries in the x-direction. A broadband (*λ* = 300–1300) source (plane-wave) with its electric field along the x-axis (axes labeled in Fig. 1(a)) was incident from within the free-space region. The absorption in each material was provided from experimental data^42,43^ and interpolated. The resulting generation rate data is exported in Matlab version R2016a (The MathWorks Inc.) where it is integrated over wavelength, using *trapz* function and subsequently plotted.

### Electrostatic simulations

We make use of SCAPS to calculate various spectral responses, e.g. $J-V$, $Q-V$, or $C-f$, for example. For each measurement performed with SCAPS, dark or light condition can be set, as well as the temperature of environment. Also, for every step wavelength and/or voltage, the screens shows the intermediate results of simulations, that can then be stored in external file. After completing the measurements it can be compared with other measurements and calculations and then saved, as well. This software is flexible, since one can either chose to specify the illumination spectrum and calculate the generation, or directly specify the generation using external data. Also, SCPAS can allow for taking into account the absorption of low energy photons (i.e. with energy lower than the bandgap) as well as band bending around the metal semiconductor interfaces due to surface states and Fermi-level pinning^52,53^.
